# Effects of variation in sample storage conditions and swab order on 16S vaginal microbiome analyses

**DOI:** 10.1128/spectrum.03712-23

**Published:** 2023-12-14

**Authors:** Tanya Kumar, MacKenzie Bryant, Kalen Cantrell, Se Jin Song, Daniel McDonald, Helena M. Tubb, Sawyer Farmer, Amanda Lewis, Emily S. Lukacz, Linda Brubaker, Rob Knight

**Affiliations:** 1 Medical Scientist Training Program, University of California San Diego, La Jolla, California, USA; 2 Department of Pediatrics, University of California San Diego, La Jolla, California, USA; 3 Department of Computer Science and Engineering, University of California San Diego, La Jolla, California, USA; 4 Center for Microbiome Innovation, Jacobs School of Engineering, University of California San Diego, La Jolla, California, USA; 5 Department of Obstetrics, Gynecology and Reproductive Sciences, University of California San Diego, La Jolla, California, USA; 6 Department of Bioengineering, University of California San Diego, La Jolla, California, USA; Wayne State University, Detroit, Michigan, USA

**Keywords:** vaginal microbiome, microbiome, sample storage, sample collection, preservation method, 16S

## Abstract

Technical bias is a pressing issue in microbiome research, and variability can be introduced at any stage from sample collection to figure generation. In this study, we aim to reduce biases in studying the human vaginal microbiome by examining the impact of sample storage buffer and multiple swabbing events using 16S rRNA gene amplicon sequencing data generated from vaginal swabs. We show that AssayAssure Genelock, a clinically relevant preservative for urine samples, is effective in preserving vaginal samples for microbiome studies. When comparing Genelock to 95% (vol/vol) ethanol and no preservative (air only), host variability explained more variance in both weighted and unweighted UniFrac measurements than the preservation method. We further examined the impact of three successive self-swabbing events, as the relatively low biomass nature of vaginal samples can inherently introduce bias. It is important to know if taking multiple swabs can provide replicable results and thus allow for additional technical replicates and an increased sample size. We found that up to three swabbing events do not introduce bias when examining the presence or absence of taxa but can explain 3% of the variability in the amount of taxa calculated. A study with more participants is warranted to provide further validation of these findings, but in producing this pilot study, we aim to continue laying the groundwork so that universally standardized and accessible studies can be created.

**IMPORTANCE:**

The composition of the human vaginal microbiome has been linked to a variety of medical conditions including yeast infection, bacterial vaginosis, and sexually transmitted infection. The vaginal microbiome is becoming increasingly acknowledged as a key factor in personal health, and it is essential to establish methods to collect and process accurate samples with self-collection techniques to allow large, population-based studies. In this study, we investigate if using AssayAssure Genelock, a nucleic acid preservative, introduces microbial biases in self-collected vaginal samples. To our knowledge, we also contribute some of the first evidence regarding the impacts of multiple swabs taken at one time point. Vaginal samples have relatively low biomass, so the ability to collect multiple swabs from a unique participant at a single time would greatly improve the replicability and data available for future studies. This will hopefully lay the groundwork to gain a more complete and accurate understanding of the vaginal microbiome.

## OBSERVATION

The vaginal microbiome plays an important role in many health conditions such as yeast infection (1), sexually transmitted infection (2) including HIV (3), preterm birth and premature rupture of membranes in pregnant individuals (4), and bacterial vaginosis (BV) (5). BV impacts an estimated 29% of females in the United States (6) and 50% of females in East/Southern Africa (7) with high relapse rates after treatment (8 – 10) of 58% (8). Standardization of sample collection procedures is necessary to improve scientific rigor and reproducibility to drive vaginal microbiome research forward. A thorough understanding of the vaginal microbiome will advance diagnoses and treatments of vaginal microbiome related health conditions.

We investigate the effects of AssayAssure Genelock (Genelock), a nucleic acid preservative designed, and shown to be effective, for urine samples (11 – 14). We compare samples preserved with Genelock to samples preserved with no preservative (air only) and 95% (vol/vol) ethanol, as ethanol has been previously shown to be an effective nucleic acid preservative (15 – 21). Additionally, we examine how swab collection order impacts the vaginal microbiome. If consecutive swabbing minimally impacts the vaginal microbiome regardless of swab order, we can strengthen sampling reproducibility and collect three technical replicate vaginal samples at a single time point.

**Fig 1:**
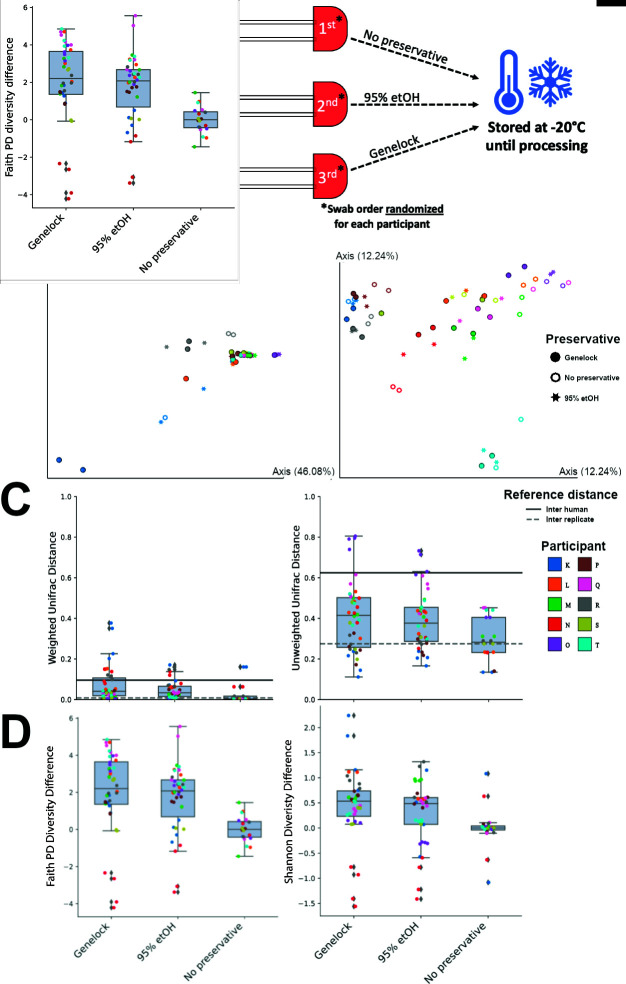
Experimental overview and data grouped by individual. (**A**) Experimental overview: Ten adult females contributed three sets of vaginal samples via dual swabs. After collection, swabs immediately went into AssayAssure Genelock (Genelock), 95% ethanol, or no preservative, then stored at −20°C until sample processing. (**B**) Principal-coordinate analysis plots of weighted and unweighted UniFrac distances grouped by individual. (**C**) Distances between each preservation method and no preservative, grouped by each participant. For example, the red dots in the Genelock bar represent the distances between the Genelock and no preservative samples from participant N while the red dots in the no preservative bar represent the distances between the no preservative replicates of participant N. (**D**) Shannon and faith PD alpha diversity differences between different preservative methods and no preservative.

Ten healthy adult females each contributed three mid-vaginal samples via self-collection under UCSD IRB protocol #801735 using cotton-tipped Falcon Double Swubes (BD), a dual swab that provided two technical replicates per collection. Immediately after collection, samples were stored in one of three preservative conditions (Fig. 1A; Text S1A) then frozen at −20°C for 24 hours until processing. Swab order was noted and randomized to minimize any potential bias impacted from preservation method. Vaginal samples and positive KatharoSeq (22) controls (Text S1B) were then aliquoted into DNA extraction bead plates and extracted using Earth Microbiome Project standard protocols (23), further updated in Shaffer et al. (24) (Text S1C). The 16S rRNA V4 region was amplified via high-throughput miniaturized PCR (25) before sequencing on an Illumina MiSeq (Text S1D). Forward read sequences were trimmed, filtered, and demultiplexed using Qiita (26) (Text S1E). Using the KatharoSeq (22) protocol, we established a limit of detection for “true” samples, allowing us to distinguish samples from trace microbes in laboratory reagents and utilized known read counts as a threshold for sample exclusion. We utilized the KatharoSeq 50% threshold, excluding three samples with less than 649 reads, and then rarefied to 30,000 reads per sample, to include 57 samples from 10 individuals (Text S1E). Eleven negative controls did not meet the rarefaction depth and did not show systematic clustering in PcoA with weighted and unweighted UniFrac (weighted PERMANOVA: *P* = 0.4822, f = 0.921; unweighted PERMANOVA: *P* = 0.7, f = 0.865).

We first examined the samples’ beta diversity metrics grouped by individual participants (Fig. 1B). In both weighted and unweighted UniFrac, permutational multivariate analysis of variance (PERMANOVA) beta diversity was driven primarily by participant (PERMANOVA, unweighted *P* = 0.001, f = 9.23; weighted *P* = 0.001, f = 12.887), rather than preservative method (unweighted *P* = 0.63, f = 0.88; weighted *P* = 0.62, f = 0.74) or swab collection order (unweighted *P* = 0.92, f = 0.66; weighted *P* = 0.58, f = 0.78). Clustering of individuals was more apparent in unweighted UniFrac, and Fig. 1B demonstrates evidence for an individual vaginal microbiota at the collection time point.

Figure 1C shows the distances between each preservation method and no preservative, grouped by each participant. The beta diversity shown in Fig. 1C reveals that UniFrac distance between the different preservation methods is below the mean distance between participants (inter-human), suggesting host as primary contributor of beta diversity. Additional multivariate analyses of variance were performed using ADONIS (27) to capture variance explained by host and preservative. Two-way comparisons were performed between Genelock vs. 95% ethanol, Genelock vs. no preservative, and 95% ethanol vs. no preservative. When comparing Genelock vs. 95% ethanol, the host accounted for more variance in both weighted and unweighted UniFrac (ADONIS: weighted, R^2^ = 0.83, *P* = 0.001; unweighted, R^2^ = 0.72, *P* = 0.001) than samples preserved in Genelock vs. no preservative (weighted, R^2^ = 0.69, *P* = 0.001; unweighted, R^2^ = 0.64, *P* = 0.001) and samples preserved in 95% ethanol vs. no preservative (weighted, R^2^ = 0.76, *P* = 0.001; unweighted, R^2^ = 0.68, *P* = 0.001). Variance explained by preservative was less when comparing samples preserved in Genelock vs. 95% ethanol (weighted, R^2^ = 0.11, *P* = 0.001; unweighted R^2^ = 0.09, *P* = 0.28, not significant) than samples preserved in Genelock and no preservative (weighted, R^2^ = 0.22, *P* = 0.001; unweighted, R^2^ = 0.16, *P* = 0.001) and samples preserved in 95% ethanol vs. no preservative (weighted, R^2^ = 0.12, *P* = 0.000; unweighted, R^2^ = 0.14 *P* = 0.001). This suggests that both Genelock and 95% ethanol may work as effective preservatives for vaginal microbiome samples, as more variance was explained by the preservative when compared to samples with no preservative. This aligns with Kumar et al. (14), where samples preserved in Genelock had little effect on the variance of urine samples when compared to urine samples preserved in 95% ethanol.

Phylogenetic and non-phylogenetic alpha diversity analyses also provide evidence that Genelock and 95% ethanol work as effective preservatives, as samples preserved by these methods had a richer diversity compared to samples with no preservative (Fig. 1D). Individual variation in Fig. 1C and 1D show that some individuals, such as participant O, have unique microbiomes that are more host-driven compared to the average participant in this cohort. We also observe that some (participant N) rank lower than average on richness, evenness, and phylogenetic-based diversity, while others (participant T) rank higher than average on phylogenetic-based diversity. Despite the small sample size, these findings further support that the vaginal microbiome is highly individualized.

**Fig 2:**
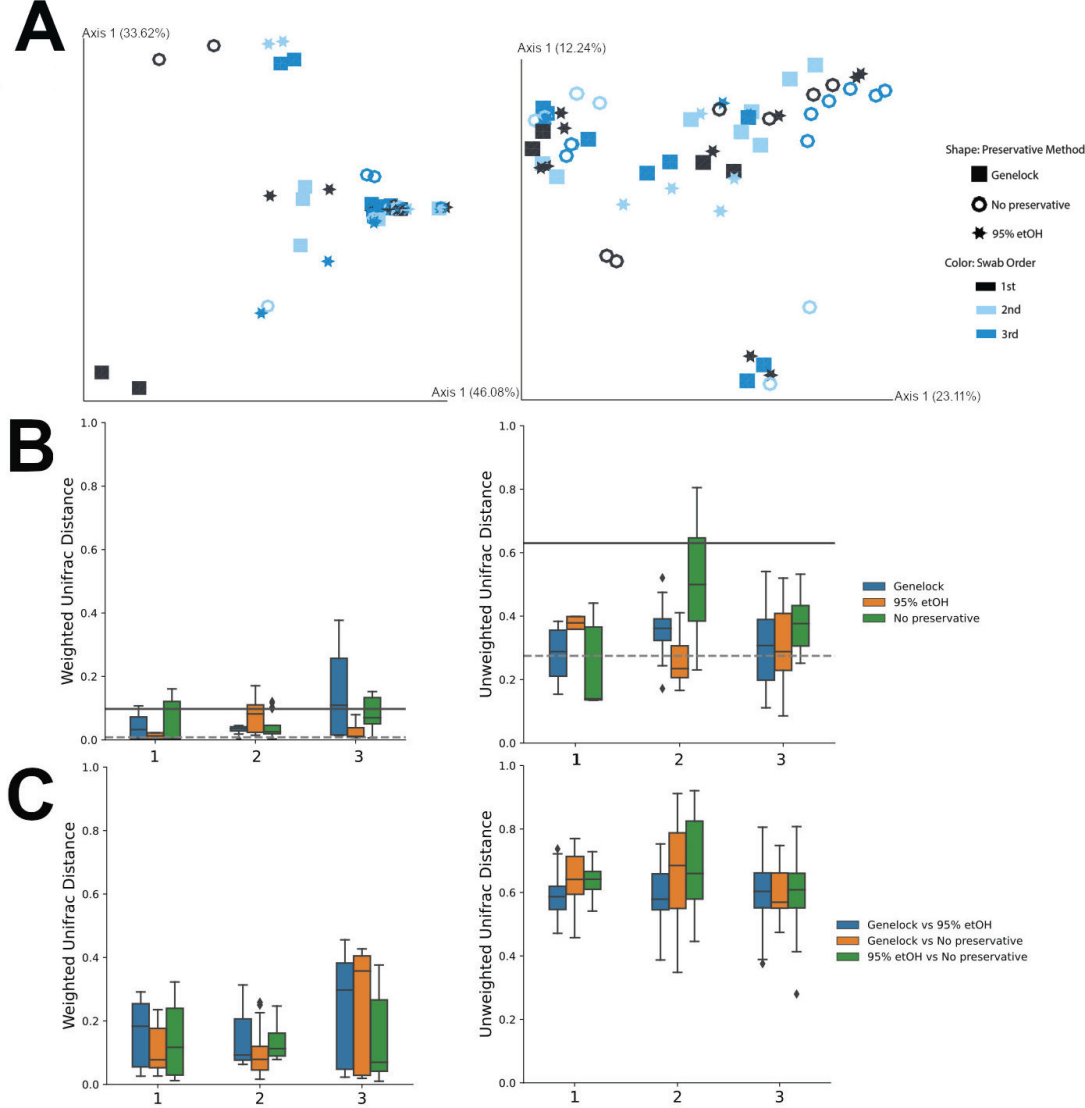
Data grouped by sample collection order. (**A**) Principal-coordinate analysis plots of weighted and unweighted UniFrac distances grouped by preservative method. (**B**) The Unifrac distance between the first swab collected from each participant and their following swabs. For example, the bars at collection point one show the UniFrac distance of the replicates for first swabs collected while the bars for swab two show the UniFrac distance between swabs 2 and 1 for each participant. (**C**) The UniFrac distance between different preservation methods and swab collection order.

Obtaining three consecutive swabs permitted analysis of collection order, which did not appear to have significant order-based clustering (Fig. 2A). There were no discernable differences in beta diversity between the first swabs collected and consecutive swabs (Fig. 2B) when considering which taxa are present (ADONIS: unweighted UniFrac, *P* = 0.358). When the amount of each taxa is considered, swab order explains approximately 3% of the variability (ADONIS: weighted UniFrac, R2 = 0.027, *P* = 0.009). This suggests that the vaginal microbiome is minimally altered when three vaginal swabs are collected consecutively. The unweighted and weighted UniFrac distances data support the beta diversity comparability of Genelock and 95% ethanol, the current laboratory standard (21), by swab order (Fig. 2C). In this small cohort, we detected minimal differences between collection order 1, 2, or 3. However, larger-scale studies with additional participants and consecutive swabbing events are warranted to confirm these findings and improve the power of the study.

Overall, our study supports the use of Genelock, as well as 95% ethanol, for vaginal swab sample storage for microbiome studies. Individual variation seems to play a more impactful role than preservation method in vaginal microbiome results, pointing towards the growing understanding of an individual vaginal microbiome. Given the possibility that swabbing order appears to have a minor effect on the vaginal microbiome, future studies may be able to incorporate additional consecutive technical replicates from individuals. Ultimately, this improves scientific rigor and reduces reproducibility concerns and sample-to-sample microbial biases that are common in microbiome research, especially in relatively lower biomass sample types including vaginal samples. Despite the clear limitations of a small sample size, this data will inform larger studies that wish to include vaginal sample collection for subsequent microbiome analyses. The pragmatic ability of research participants to self-collect vaginal samples, augmented with robust evidence for sample storage and microbiome analysis, holds great promise for advancing obstetric and gynecologic research in the near future.

## Data Availability

The data generated in this study are available publicly in Qiita under study ID 14385 (https://qiita.ucsd.edu/public/?study_id=14385). Sequence data has been deposited at EBI/ENA under accession number ERP138440. A STORMS checklist (28) is available for this study (https://doi.org/10.5281/zenodo.7439328).

## SUPPLEMENTAL MATERIAL

Fig. S1 spectrum.03712-23-s0001.eps Class-level taxonomic analysis.

Fig. S2 spectrum.03712-23-s0002.eps Beta diversity analysis of blanks.

Supplemental text spectrum.03712-23-s0003.pdf Text S1A to S1E; legends for Fig. S1 and S2.

ASM does not own the copyrights to Supplemental Material that may be linked to, or accessed through, an article. The authors have granted ASM a non-exclusive, world-wide license to publish the Supplemental Material files. Please contact the corresponding author directly for reuse.
