# Supplementary material for: Effects of variation in sample storage conditions and swab order on 16S vaginal microbiome analyses
Source: Microbiol Spectr. 2023 Dec 14;12(1):e03712-23. doi: 10.1128/spectrum.03712-23 (PMC10783137; doi:10.1128/spectrum.03712-23)
Supplement: Supplemental text — Text S1A to S1E; legends for Fig. S1 and S2. [file spectrum.03712-23-s0003.pdf]

Text S1 - Supplemental Materials and Methods

A) Volunteer Recruitment and Sample Collection

a) Volunteers, composed of healthy female adults, were recruited via word of mouth in the Knight Lab at Biomedical Facility II on UC San Diego Health campus (La Jolla, CA). "Healthy" was defined by the participant not displaying any active symptoms of disease such as fever, chills, cough, shortness of breath, headache, loss of taste or smell, sore throat, congestion, nausea or vomiting, or diarrhea. Volunteers gave written consent (UCSD IRB protocol #801735) after being provided with and reading through the consent document.

b) Ten volunteers donated 3 sets of mid-vaginal dual swabs, collected on sterile cotton-tipped Falcon Double Swubes (BD) via self-swabbing. After collection, swabs were stored with AssayAssure® Genelock (Sierra Molecular) or 95% (v/v) ethanol, ensuring that the head of the swab was completely covered, or with no preservative. Dual swabs were not separated and always received the same preservation method. The order of swab collection (1st, 2nd, 3rd) was randomized based on intended preservative condition and noted to investigate if the microbial diversity changes over the course of swabbing events, indicating whether or not we can obtain multiple vaginal mucus samples from the same participant at the same time point. Samples were then frozen immediately at -20°C (**Figure 1A**). Each preservative condition had 2 technical replicates per participant.

B) KatharoSeq Isolate Growing

a) The KatharoSeq (1) positive control strains were *Bacillus subtilis,* a Gram positive bacterium, and *Paracoccus denitrificans,* a Gram negative bacterium. *B. subtilis* and *P. dentrificans* are commonly found in the soil and not typically in human and vaginal microbiome

samples, making them appropriate controls. The two strains were grown independently at 37°C and harvested at the log stage. Cells were counted using standard microbiology methods and then mixed together to act as one control. The mock community control was then serially diluted to obtain a range of known cell counts.

C) Plating and extraction

a) Samples were plated and extracted using the Earth Microbiome Project standard protocols (2), as updated in Shaffer, et al. (3). After taking vaginal mucus samples out of -20°C storage and thawing, swabs were plated into 96-well MagMAX Microbiome Bead Plates (ThermoFisher Scientific). Additionally, eight serially diluted Katharoseq positive controls (*Paracoccus denitrificans*. and *Bacillus subtilis*) were plated with cell counts ranging from 248,000 to 0 cells (1). Negative controls consisted of unused dual swabs that contained no vaginal sample and were placed in either Genelock, 95% ethanol, or no preservative. Additional negative controls consisted of extraction blanks, which contained no swabs (used or unused) and consisted of extraction reagents only. After the addition of MagMAX Microbiome Lysis Solution (ThermoFisher Scientific), samples were bead beaten for 2 minutes and extracted using the MagMAX Microbiome Ultra NA Extraction Kit (ThermoFisher Scientific).

D) 16S rRNA Amplification and Sequencing

a) Following extraction, amplification of the 16S rRNA V4 region was performed via a high-throughput miniaturized PCR protocol (4). Unique 515f-806r Golay barcodes (2) were used in order to allow for post-sequencing demultiplexing. After library amplification, libraries were equal volume pooled, PCR cleaned (QIAGEN Cleanup Kit), quantified, diluted to a loading

concentration of 16pM, and loaded on an Illumina MiSeq using a MiSeq 300 cycle (2x151 paired end reads) reagent kit (v2) at the University of California, San Diego for sequencing. All 16S amplicon PCR amplification and sequencing protocols adhered to Earth Microbiome Project standard protocol (2), as updated in Minich, et al. (4).

E) Post-Sequencing Processing

a) Forward read sequences generated from the MiSeq were trimmed to 150 nucleotides, quality filtered, and demultiplexed using Qiita (5). The KatharoSeq 50% threshold to exclude any samples with less than 649 reads and then rarefied to 30,000 reads, resulting in a pool of 57 samples and 10 individuals.

b) Data can be found on Qiita study #14385 (https://qiita.ucsd.edu/public/?study_id=14385), and has been deposited at EBI/ENI under EBI accession ERP138440 (https://www.ebi.ac.uk/ena/browser/view/PRJEB53632).

References:

1. Minich JJ, Zhu Q, Janssen S, Hendrickson R, Amir A, Vetter R, Hyde J, Doty MM, Stillwell K, Benardini J, Kim JH, Allen EE, Venkateswaran K, Knight R. 2018. KatharoSeq Enables High-Throughput Microbiome Analysis from Low-Biomass Samples. mSystems 3:e00218-17.

2. Earth Microbiome Project. 2023. Earth Microbiome Project Protocols and Standards.

3. Shaffer JP, Marotz C, Belda-Ferre P, Martino C, Wandro S, Estaki M, Salido RA, Carpenter CS, Zaramela LS, Minich JJ, Bryant M, Sanders K, Fraraccio S, Ackermann G, Humphrey G, Swafford AD, Miller-Montgomery S, Knight R. 2021. A comparison of DNA/RNA

extraction protocols for high-throughput sequencing of microbial communities. BioTechniques 70:149–159.

4. Minich JJ, Humphrey G, Benitez RAS, Sanders J, Swafford A, Allen EE, Knight R. 2018. High-Throughput Miniaturized 16S rRNA Amplicon Library Preparation Reduces Costs while Preserving Microbiome Integrity. mSystems 3:e00166-18.

5. Gonzalez A, Navas-Molina JA, Kosciolek T, McDonald D, Vázquez-Baeza Y, Ackermann G, DeReus J, Janssen S, Swafford AD, Orchanian SB, Sanders JG, Shorenstein J, Holste H, Petrus S, Robbins-Pianka A, Brislawn CJ, Wang M, Rideout JR, Bolyen E, Dillon M, Caporaso JG, Dorrestein PC, Knight R. 2018. Qiita: rapid, web-enabled microbiome meta-analysis. Nat Methods 15:796–798.

6. Minich JJ, Sanders JG, Amir A, Humphrey G, Gilbert JA, Knight R. 2019., Quantifying and Understanding Well-to-Well Contamination in Microbiome Research. mSystems 4:e00186-19.

Figure Legends:

Supplemental Figure S1: Class-level taxonomic analysis. For most subjects, the compositional differences in duplicate swabs are visually consistent, whereas the differences between preservatives is quite noticeable. This is with the exception of subject K, where we suspect one of the duplicate swabs may have been slightly contaminated on collection.

Supplemental Figure S2: Beta diversity analysis of blanks. (A) Unweighted and (B) Weighted UniFrac distances between blanks and vaginal samples show that all blanks have significant grouping away from the vaginal samples, indicating that contamination is unlikely. Statistical

significance is supported by paired PERMANOVA values for (C) Unweighted and (D) Weighted UniFrac distances. Nonetheless, well-to-well contamination is always a possibility, which has been previously explored (6).
